# Encoding and decoding time in neural development

**DOI:** 10.1111/dgd.12257

**Published:** 2016-01-09

**Authors:** Kenichi Toma, Tien‐Cheng Wang, Carina Hanashima

**Affiliations:** ^1^ Laboratory for Neocortical Development RIKEN Center for Developmental Biology Kobe 650‐0047 Japan; ^2^ Department of Biology Graduate School of Science Kobe University Kobe 657‐8501 Japan

**Keywords:** cell cycle, neural stem cell, temporal code, timing, transcriptional network

## Abstract

The development of a multicellular organism involves time‐dependent changes in molecular and cellular states; therefore ‘time’ is an indispensable mathematical parameter of ontogenesis. Regardless of their inextricable relationship, there is a limited number of events for which the output of developmental phenomena primarily uses temporal cues that are generated through multilevel interactions between molecules, cells, and tissues. In this review, we focus on neural stem cells, which serve as a faithful decoder of temporal cues to transmit biological information and generate specific output in the developing nervous system. We further explore the identity of the temporal information that is encoded in neural development, and how this information is decoded into various cellular fate decisions.

## Graphical abstract

This review focus on neural stem cells as a decoder of temporal cues to transmit biological information and generate particular output in the developing nervous system.

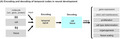

## Introduction

If ‘time’ is defined through the passage of events and changes in states, *development*, or the formation of a multicellular organism from a single fertilized egg, can be considered one of the most dynamic times one could perceive in nature. Conversely, development itself can be interpreted as a system based on the readout of multiplex temporal information. In this review, we explore the identity of the temporal information that is encoded in the system and how this information is decoded as cellular activity to generate specific outputs, based on *in vivo* and *in vitro* neural development models.

### What is ‘time’ in development?

**Figure 1:**
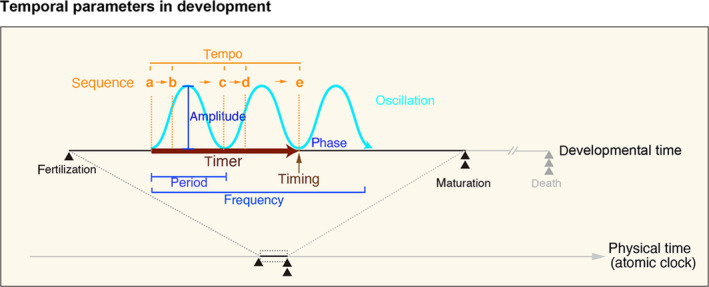
Temporal parameters used in development. The entire developmental time scale period can be plotted onto a physical time scale. Along this axis, the timer (bold brown arrow) determines the timing (thin brown arrow) of a specific developmental event (i.e. event ‘e’) relative to a reference event (i.e. event ‘a’) along the directional temporal axis. Temporal cues are also produced through periodic events such as oscillations (light blue). In this case, the amplitude, phase, period or frequency (blue) may serve as temporal codes.

What aspects of ‘*time’* are used in the developmental process? Here, we first define the conceptual terms related to time within the context of development to expand our discussion in the following sections (Box 1). Let us plot the entire development period onto a physical time scale (Fig. 1). Along this axis, the timer determines the timing of a specific developmental event relative to a reference event along the directional temporal axis (Fig. 1, ‘*timer*’ and ‘*timing’*). Under normal circumstances, the time intervals between two or more developmental events are reproducible within individual species with little deviation; therefore, such durations can be measured and predicted, in theory, using the atomic clock (Fig. 1, ‘*physical time’*). However, unlike physical clock‐defined events (i.e., the swing of a pendulum or the motion of planets; although one can also consider universal gravitation as a relativity factor), the timing of developmental events is determined by temporal cues that are generated within the system itself; therefore, if the timer is set early or with a delay, it will likely shift subsequent events relative to the reference event. In this review, we will discuss the molecular entities of such ‘developmental timers’ as well as how they are decoded into various cellular fate decisions by focusing on neural stem cells.

Box 1 **Temporal terms in development**

**Table:** 

Time	An abstract mathematical parameter that is perceived through the recognition of state changes in development.
Time scale	A measurable dimension of time from fertilization to maturity that records the passage of events and their intervals.
Clock	A high‐precision timekeeper based on atomic clocks that is used to measure absolute developmental time relative to other biological and physical events.
Timer	A device that measures time interval(s) between two or more sequential developmental events.
Timing	The control of the occurrence of events, pace, or period over time scales to achieve reproducible and coordinated output of developmental phenomena.
Oscillation	A periodic change between two or more molecular/cellular/tissue states that is typically achieved through positive or negative feedback loops. Oscillation cycles are defined by amplitude and phase.
Frequency	The number of occurrences of a recurring event within a given unit of time (if per one second = Hz).
Period	Duration of one cycle of a recurring event. In a broader context, it can refer to the length of a specific event on a linear time scale.
Phase	A characteristic of a cycle relative to the beginning of a period.
Amplitude	The maximum value of a variable during a defined period.
Tempo	The relative velocity or pace of events that occur within a defined time scale.

## Decoding time: neural stem cells as a readout of temporal codes

As mentioned previously, development and time are inextricable in the sense that any given phenomenon concerning ontogenesis involves a time‐dependent change in molecular or cellular states. However, regardless of their inseparable relationship, there is a limited number of events for which the output of developmental phenomena primarily exploits temporal cues that are generated through multilevel interactions between ions, RNA, proteins, cells, and tissues. In this section, we refer to how neural stem cells serve as a faithful decoder of temporal cues to transmit biological information and generate particular output in the developing nervous system.

**Figure 2:**
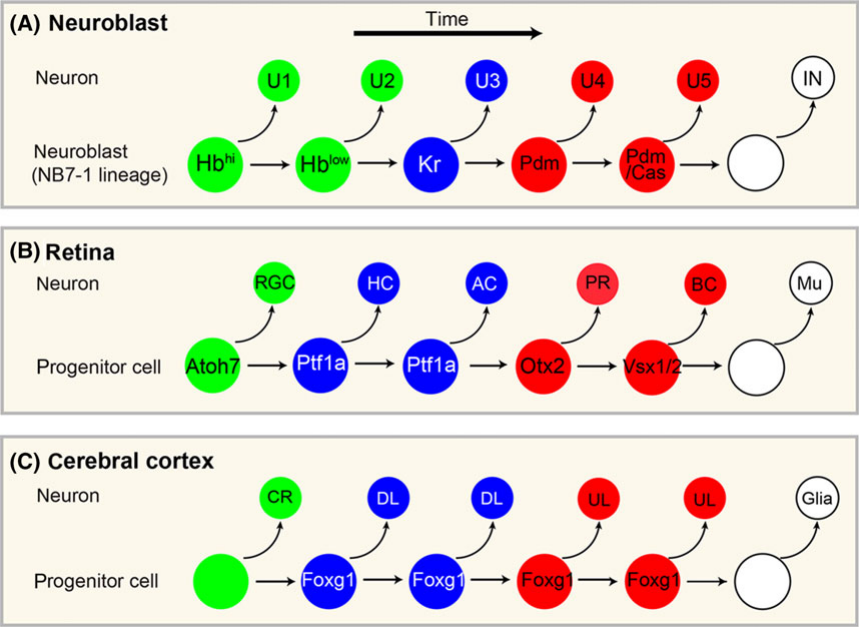
Temporal cell types in various neural systems. (A) In Drosophila N7‐1 lineage, neuroblasts expresses distinct transcription factors, Hb, Kr, Pdm and Cas to regulate U1‐U5 neuron specification and temporal identity transitions. (B) In retina, specification of subtypes is encoded in part through expression of transcription factors. Photoreceptor cells (PR) compose of cone and rod cells, in which cone cells are generated at an earlier time window than rod cells. (C) In the cerebral cortex, neural stem (progenitor) cells produce Cajal‐Retzius (CR) cells, deep‐layer (DL) neurons and upper‐layer (UL) neurons sequentially. Foxg1 has been shown as a temporal identity factor that switches neural stem cell competence from CR cells to DL neurons. AC, amacrine cells; BC, bipolar cells; HC, horizontal cells; Mu, muller glia cells; PR, photoreceptor cells; RGC; retinal ganglion cells.

Our conceptual basis of ‘temporal decoding’ was advanced by studies of three independent neural systems. Birthdating and cell lineage studies revealed that in the *Drosophila* nerve cord (Bossing *et al*. 1996; Schmid *et al*. 1999), the retina (Carter‐Dawson & LaVail 1979; Young 1985; La Vail *et al*. 1991) and the cerebral cortex (Angevine & Sidman 1961; Rakic 1974), neural stem cells (which in this broad context include neural progenitor cells and neuroblasts that have the capacity to self‐renew through symmetric or asymmetric cell divisions) exhibit a highly reproducible temporal order of the production of distinct cell types that compose mature neural circuits. In *Drosophila* embryos, ventral nerve cord neuroblasts sequentially produce U1 through U5 neurons, which are identified by the expression of the transcription factors Hunchback (Hb), Kruppel (Kr), Pdm, Castor (Cas), and Grainyhead (Grh). The generation of U1–U5 neurons is followed by the production of interneurons (Fig. 2A). In the retina, retinal progenitor cells (RPCs) generate different cell types in the following sequence: retinal ganglion cells (RGC), horizontal cells (HC), amacrine cells (AC), photoreceptor cells (PR), bipolar cells (BC) and Muller glia cells (Mu) (Rapaport *et al*. 2004) (Fig. 2B). In the cerebral cortex, neural progenitor cells also produce distinct neuronal subtypes in the temporal order: Cajal‐Retzius (CR) cells and subplate neurons, deep‐layer (DL) subcortical projection neurons, upper‐layer (UL) intracortical projection neurons (Fig. 2C). The output of distinct neuronal subtypes along the temporal axis created the concept of a ‘developmental timer’ that are encoded within the cells, which measures the time intervals to switch the production of neurons from one type to the next.

To test the entity of such a developmental timer, the first experiment performed was to assess the localization of the code by heterochronic transplantation (McConnell & Kaznowski 1991; Desai & McConnell 2000), in which neural progenitor cells of donor ferret cortex were isolated and transplanted into the progenitor zones of recipient ferrets of heterochronic developmental stages. These studies revealed that early‐born neural progenitor cells can sense the ‘aged’ environment and acquire later‐born UL cell identities when transplanted into an environment in which time has elapsed; however, later‐born neural progenitor cells lose the ability to respond to such cues, even when they are transplanted into a temporally younger environment. These pioneering studies provided the conceptual basis that both the extrinsic cues, as well as the intrinsic changes in the capacity of neural stem cells to respond to such cues, can constitute the developmental timer. This concept has also been raised in neural stem cells outside the cerebral cortex, in which retinal progenitor cells (RPCs) and *Drosophila* neuroblasts (Rapaport & Dorsky 1998; Berger *et al*. 2001) also follow a strict rule according to which subtypes are produced in a defined temporal sequence. With these advantages, neural stem cells serve as a unique processing unit in which temporal measurements (time progression) and output (neuronal types) are tightly correlated. In the following sections, we decipher where and how the temporal information that controls the behavior and output of neural stem cells is encoded.

## Encoding of time: lessons from *in vitro* systems

**Figure 3:**
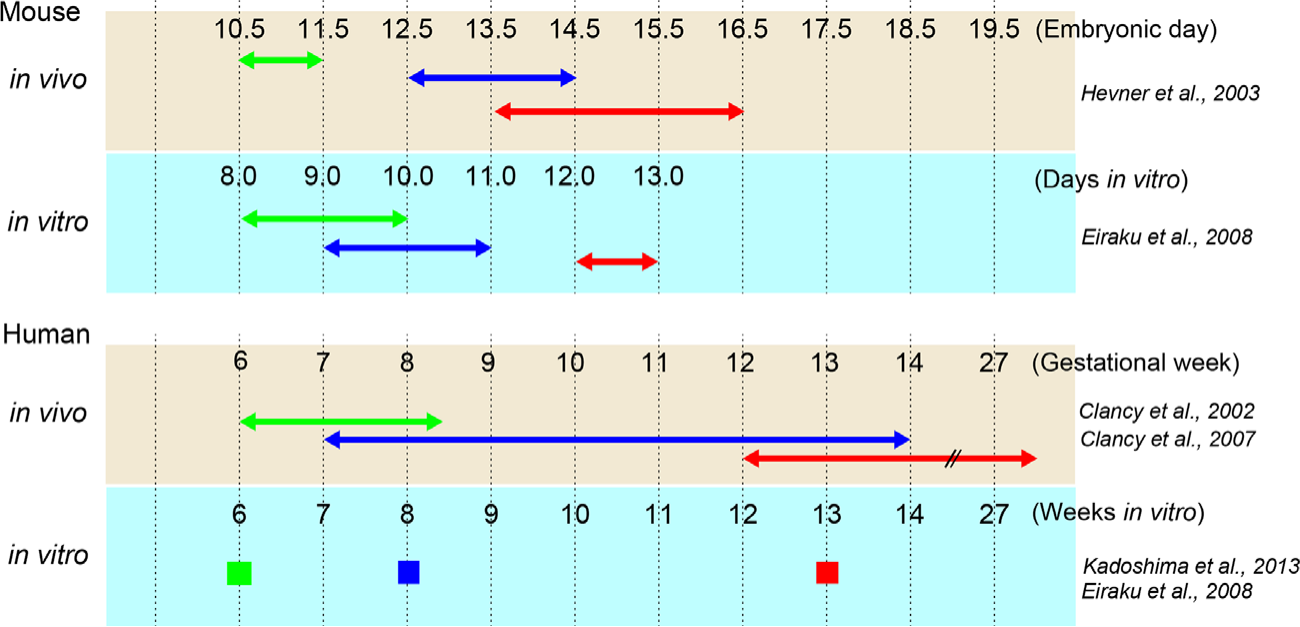
Comparison of the timing of neurogenesis between *in vivo* and *in vitro* models. *In vivo* and *in vitro* neurogenesis of mouse (top panels) and human (bottom panels) cortical stem cells reveal similar temporal scale in neuronal subtype production. *In vitro* time scale is calculated based on the days (mouse) or weeks (human) of culture. For human *in vitro* data, only limited time points have been observed and the start and end period of each subtype neurogenesis has not been determined. CR cell (green), deep‐layer neuron (blue), upper‐layer neuron (red).

Where is ‘time’ encoded during neural development? As an entry point, one can refer to *in vitro* models in which isolated progenitor cells from the mouse cerebral cortex were subjected to single cell culture (Shen *et al*. 2006) or induced from mouse and human embryonic stem cells (ES cells) (Shen *et al*. 2006; Eiraku *et al*. 2008; Gaspard *et al*. 2008; Kadoshima *et al*. 2013). Induced cortical cells were cultured and monitored for subsequent development; these cells exhibited a highly reproducible sequence of cell type generation as observed *in vivo*. Among several culture conditions, three‐dimensional aggregate culture (suspension of mouse and human ES cells with quick reaggregation, which results in the formation of embryoid body‐like tissues), is currently the most effective method to maintain long‐term cultures that exhibit the neurogenesis and apicobasal cortical polarity that mimics endogenous development (Eiraku *et al*. 2008, 2011; Kadoshima *et al*. 2013; Lancaster *et al*. 2013). These models have provided several critical insights concerning organogenesis: (i) self‐organization of neural stem cells occurs in the absence of surrounding tissues (Eiraku *et al*. 2008, 2011; Nakano *et al*. 2012; Kadoshima *et al*. 2013); (ii) the generation intervals between sequential neuronal subtypes are surprisingly similar to the timing of neurogenesis observed *in vivo* (Fig. 3); (iii) the timing with which neuronal subtypes are generated from neural stem cells is inherent to the neurogenesis tempo observed in normal mouse and human development (Fig. 3); and (iv) the ultimate size of the tissue produced is proportional to that expected based on species tissue size. In these organoids, the initial cell aggregate is composed of 3000–9000 ES cells and lacks long‐range inter‐organ signals such as suprachiasmatic nucleus‐mediated circadian rhythms. The fact that neural stem cells can accurately sense time in these isolated conditions argues that the ‘temporal code’ is generated within the organoids themselves. Furthermore, differences in the durations of periods of tissue formation and the time intervals required to produce distinct neuronal subtypes in ES cells derived from mice and humans indicates that the temporal code depends on parameters that differ between mouse and human cells. Below, we will assess each of these parameters to examine the molecular mechanisms of temporal encoding in neural stem cells.

## Periodicity‐driven temporal codes

**Figure 4:**
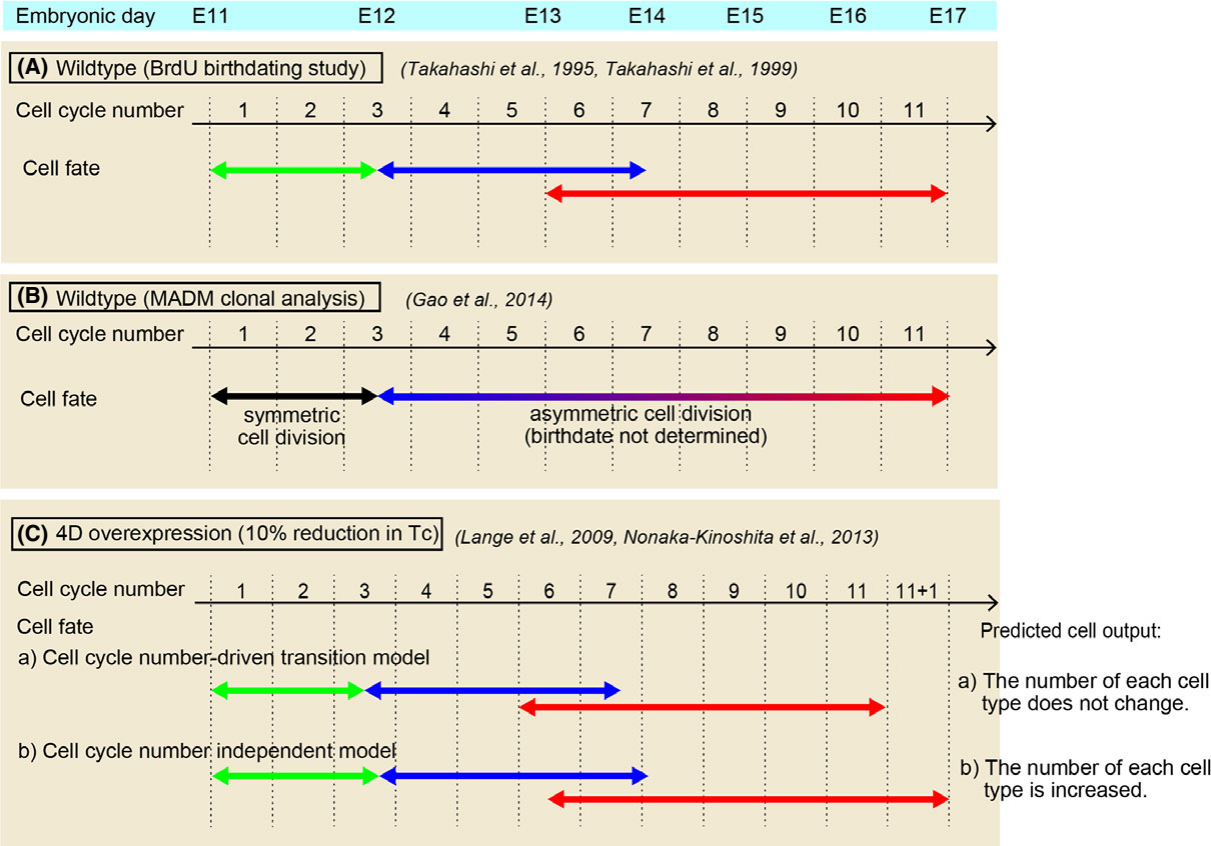
Correlation between cell cycle number and neuronal output in the cerebral cortex. (A) Based on BrdU and thymidine double‐label birthdating studies in mice (Takahashi *et al*.^1999^), cortical progenitor cells undergo 11 cell cycles after embryonic day (E)11, which sequentially produce CR cells, deep‐layer neurons and upper‐layer neurons. (B) MADM clonal analysis (Gao *et al*. 2014) reveals that cortical progenitor cells undergo an average of 2.6 symmetric cell divisions followed by 8–9 cell divisions, resulting in a similar number of total cell cycles as in study (A). (C) Manipulation of cell cycle through overexpression of Cdk4 and CyclinD (4D) results in 10% reduction in total cell cycle length. If neural progenitor cells use number of cell cycles as timer, then the prediction is that each temporal transitions are accelerated; however, the number of each cell types does not change (a). If temporal identity transitions progress independently of cell cycle (cell cycle independent timer) then the number of each cell type is expected to increase (b). 
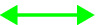
 CR cell, 
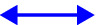
 Deep‐layer neuron, 
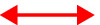
 Upper‐layer neuron.

The *in vitro* data suggest that the timing of neuron production and tissue size are encoded at the level of individual cells and their interactions. Keeping this in mind, detailed examination of neural stem cells *in vivo* has provided critical insight into the mechanisms by which their output is controlled during neurogenesis. Gao and colleagues carried out quantitative clonal analysis of mouse cortical progenitor cells, in which the progenies of an *Emx1* ^*Cre/+*^ or *Nestin‐Cre* lineage were subject to MADM analysis, which enables tracking of the fate and number of each clone that arises from the first two daughter cells produced by a single mother progenitor cell (Gao *et al*. 2014) (Fig. 4B). This analysis revealed two fundamental rules governing temporal cell behavior *in vivo*: (i) while at population levels, progenitor cells gradually switch from symmetric to asymmetric cell divisions, each clone exhibits variable timing in switching from self‐renewal (symmetric division) to neurogenic (asymmetric division) mode; and (ii) despite these temporal fluctuations, following entry into the asymmetric division phase, each neurogenic clone typically produces 8–9 cells (mean of 8.4 cells), with similar proportions of deep‐ and upper‐layer subtypes (Gao *et al*. 2014). They also identified that while the transition from symmetric to asymmetric division occurred predominantly at E11–E12, neural progenitor cells at E11 typically underwent approximately 2.6 rounds of division before entering the neurogenic phase (Gao *et al*. 2014) (Fig. 4B). If we take all types of cell division into account (i.e., cells that divide apically or basally, which is observed in most mammalian species), then an average of 8.4 neurons will arise from 8.4 asymmetric cell divisions (i.e., in the case that the last progeny enter the gliogenic phase; if the last progeny undergo terminal neurogenesis, then 7.4 divisions). This number, 11 (8.4 asymmetric + 2.6 symmetric), surprisingly mimics the number of cell cycles that cortical progenitor cells at E11 were found to undergo in 2‐h cohort BrdU‐^3^H‐TdR birthdating studies (Takahashi *et al*. 1999) (Fig. 4A). Thus, it appears that the number of cell divisions and their output is strictly determined within the clones.

### Cell cycle oscillation

**Figure 5:**
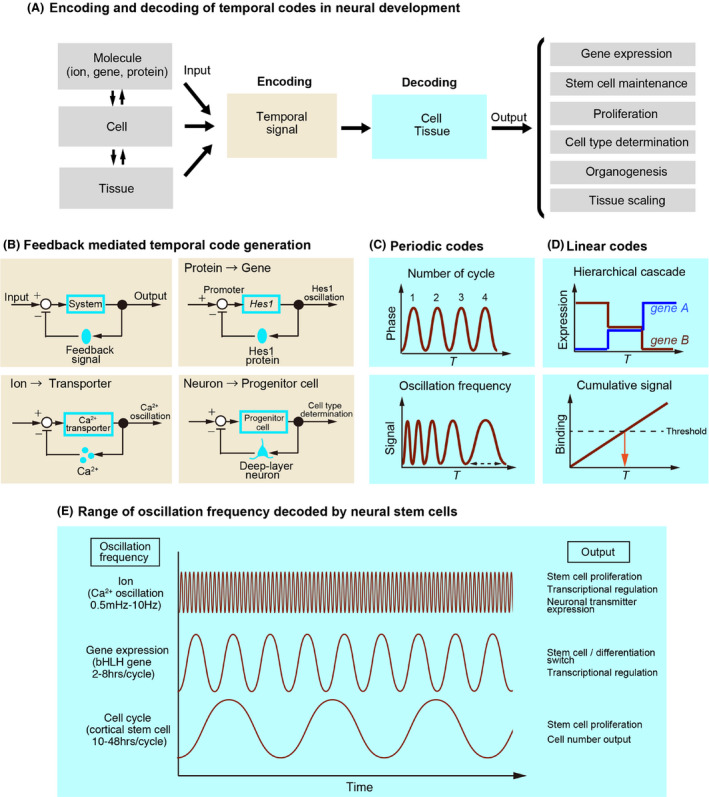
Temporal codes utilized in neural development. (A) Encoding and decoding of temporal codes in neural development. Interplay between molecules, cells and tissues generate temporal signals that are decoded by cells or tissues to generate specific outputs. (B) Examples of temporal codes that are generated through negative feedback signaling. Protein‐to‐gene and ion‐to‐transporter feedback generates periodic signals (oscillation), neuron‐to‐progenitor cell feedback triggers switch in cell types. (C) Distinct parameters of periodic signals can be used as temporal codes, such as number of cycles or oscillation frequency. (D) Linear temporal codes. Hierarchical cascade of transcription factors triggers sequential gene expression and transition in cell states. Cumulative signals can act as threshold‐driven timer to trigger temporal events. (E) Various oscillation encoded temporal cues in neural development. Oscillations are observed at ion, gene and cell levels, which are decoded into distinct cellular outputs.

How is such quantitative information encoded at clonal levels and then decoded to restrict cell number and fate? Given the stereotypic number of cell divisions that neural stem cells undergo following commitment to neurogenesis, it is reasonable to speculate that the cell cycle itself may serve as a ‘clock’ to keep track of elapsed time in stem cells and their progeny. In such cases, two parameters could possibly contribute to the counting of time in neural stem cells: the number of cell cycles determines each cell fate transition, or the length of the cell cycle period (frequency) acts as a threshold to trigger fate transition (Fig. 5C). In theory, both of these parameters can be tested by manipulating the tempo of the cell cycle *in vivo*.

If the timing of neurogenesis is determined solely by the number of cell divisions, then each cell division should be tightly coupled to the output of a given cell type. In such cases, manipulating the cell cycle period can either accelerate or decelerate the timing of each transition. To accelerate the frequency of the cell cycle, an experiment was conducted in which neural stem cells in the mouse neocortex overexpressed cyclin and cyclin kinase Cdk4/CyclinD (4D) from the onset of neurogenesis (E11.5) to late neurogenesis (E15.5) (Nonaka‐Kinoshita *et al*. 2013) (Fig. 4C). Overexpression was removed at E15.5, and at P21, the cortex revealed a significant increase in thickness and number of neurons compared with these values for normal development. Importantly, these manipulations did not affect the overall ratio of neuronal subtypes; the relative proportions of deep‐ and upper‐layer neurons appeared to be conserved. If cell division is tightly coupled to output (deep‐ or upper‐layer neurons), the number of cell cycles required for transitions should be fixed. Therefore, this should not affect the ultimate number of neurons of specific subtypes; instead, it should simply result in early termination of neurogenesis (Fig. 4C, model a). Therefore, these results indicated that the cell cycle and timing of transitions are not coupled (Fig. 4C, model b), suggesting that cell cycle number cannot act as a temporal code to control the differentiation of neural stem cells. However, it remains possible that in these mice, the increase in cell number results from expansion of the symmetric division period rather than an increase in the number of cells arising from asymmetric cell division; in the future, the combination of manipulations of the cell cycle in a clone‐restricted manner with birthdating experiments would likely provide further insight into the behavioral output of neural stem cells in these studies.

Let us consider the second possibility that the cell cycle period itself may function as a timer. In this view, an increase in the length of the cell cycle over the course of the neurogenesis period in the cerebral cortex (Tc 7.5 h at E11, 15 h at E14, and approximately 20 h at E16 in mouse) (Takahashi *et al*. 1995) may trigger the switch to neurogenesis. In this view, when the cell cycle period reaches a certain threshold, the differentiation potential of neural stem cells is switched (Fig. 5C, ‘*oscillation frequency*’). This hypothesis is also consistent with the observation that the G1 phase of the cell cycle is specifically lengthened in neural stem cells that are committed to exit the cell cycle (Arai *et al*. 2011). In 4D cell cycle manipulations (Fig. 4C), forced acceleration of the cell cycle mimics the status of the early corticogenesis period, suggesting that the output of neurons could be biased towards the early DL fate (Fig. 4A). However, the results instead indicated that UL neurons are similarly increased, with no obvious changes in the deep‐ to upper‐layer neuron ratio (Nonaka‐Kinoshita *et al*. 2013). Similarly in the retina, clonal analysis using retinal progenitor cells from E20 rat embryos and video recording revealed that for the retinal neuronal subtypes (AC, PR, BC, Mu), there were no significant correlations between the cell cycle length and the output of specific neuronal types (Gomes *et al*. 2011). Taken together, these studies suggest that the fate of neurons can be largely uncoupled from cell cycle oscillations in multiple neural developmental systems. It remains possible, however, that the specific phase of the cell cycle has implications for the coding of neurogenesis timers in stem cells. In the aforementioned 4D overexpression system, the overall reduction in the cell cycle period was approximately 10%; however, the relative length of each phase was more severely affected (S phase: 35% increase, G2‐M phase: 25% increase, G1 phase: 30% decrease) (Lange *et al*. 2009). Thus, it is possible that the period of a specific phase of the cell cycle is directly correlated with the temporal output of neurogenesis. Indeed, such a mechanism has been reported in ES cells, in which the G1‐S phase of the cell cycle is particularly prone to differentiation cues (Gonzales *et al*. 2015). When this G1‐S phase is shortened, ES cells exhibit resistance to differentiation cues to maintain their pluripotency (Burdon *et al*. 2002), indicating direct coupling of the cell cycle phase with cell differentiation machinery.

While fate transitions between different cell types may use alternative temporal codes, it is also apparent that the cell number output within a given temporal window is directly correlated with cell cycle frequency. In cynomolgus monkeys, the number of cells in area 17 is substantially higher than that in area 18 (Rockel *et al*. 1980). This is in part due to the higher rate of cell cycle re‐entry and the shorter G1 phase found in neural stem cells of area 17 compared to those in area 18 (Lukaszewicz *et al*. 2005).

### Gene oscillation

In addition to the periodic cycles of cell division, it has been shown that gene oscillation also has an instructive role in determining the behavior of neural stem cells. Monitoring expression dynamics using highly sensitive real‐time imaging of reporter‐fused proteins, revealed that bHLH transcription factors display gene‐specific periodic expression (Fig. 5E). In neural stem cells, Hes1 (Fig. 5B, top right) and Ascl1 (Mash1) oscillate with a 2‐ to 3‐h period, and Olig2 expression oscillates with a 5‐ to 8‐h period. When these oscillations are cancelled via stable expression of one of the three bHLH transcription factors, neural stem cells proceed to astrocyte (Hes1), neuron (Ascl1), and oligodendrocyte (Olig2) differentiation (Imayoshi *et al*. 2013; Imayoshi & Kageyama 2014). Manipulating the oscillation frequency of Ascl1 using light‐switchable transactivator‐mediated gene expression revealed that the 3‐h oscillation cycle of Ascl1 was sufficient to induce self‐replication in neural stem cells, whereas constitutive photoactivation resulted in stabilized Ascl1 expression and triggered neuronal differentiation (Imayoshi *et al*. 2013). These results suggest that the oscillation frequency of transcription factor proteins acts as a critical timing component responsible for switching neural stem cells to differentiation (Fig. 5C, E). Because the oscillation frequencies of bHLH genes are faster than the cell cycle period (Fig. 5E), it is unlikely that the cell cycle itself controls gene oscillations; however, it is also recognized that in cultured neural stem cells, the two daughter cells acquire distinct phases and frequencies in gene oscillation after each cell division (Shimojo *et al*. 2008). Furthermore, Hes1 gene oscillation is dependent on culture cell density; low‐density culture was shown to result in a 60–85% reduction of Hes1 oscillation due to a reduction in cell‐cell contact‐mediated Notch signaling (Shimojo *et al*. 2008). These observations leave the possibility that contact‐dependent signals may account for both cell cycle and gene oscillation in neural stem cells. In the future, experiments that assess gene oscillation patterns in cell cycle‐arrested conditions will allow assessment of epistasis between molecular and cellular periodicity events, and extraction of the critical parameters that contribute to neural stem cell maintenance and neuronal subtype differentiation.

### Calcium oscillation

Neuronal activity plays a fundamental role in neuronal maturation and neural circuit assembly; however, neural stem cells are also susceptible to patterns of correlated activity during early developmental stages. Calcium ions (Ca^2+^) act as efficient mediators of signal transduction due to the low intracellular (100 nmol/L) and high extracellular concentrations (approximately 1 mmol/L) present at resting cell states. With this disparity, a small Ca^2+^ increase within the cytoplasm can effectively trigger diverse responses with variable speeds (microseconds up to hours) and amplitudes. Furthermore, such Ca^2+^ increments occasionally elicit spontaneous oscillations of Ca^2+^ at the single‐cell level; these oscillations are propagated across multiple cells to initiate correlated temporal patterns. The temporal coding of repetitive Ca^2+^ changes is mediated through distinct extracellular stimuli, which can induce a range of oscillation frequencies in different neural cell types (Koester & Sakmann 2000; Ikeda *et al*. 2003; Imayoshi *et al*. 2013). These Ca^2+^ waves are triggered by the influx and efflux of Ca^2+^, followed by the coordination of multiple cellular transporters that act to restore equilibrium (Fig. 5B). As part of this process, interactions between Ca^2+^ spikes and downstream signaling establish oscillations of various frequencies (Gorbunova & Spitzer 2002) (Fig. 5E). Importantly, these Ca^2+^ waves can also be triggered in cortical organoids *in vitro* (Eiraku *et al*. 2008; Lancaster *et al*. 2013), indicating that spontaneous Ca^2+^ oscillations do not require exogenous triggers outside the neural stem cell derivatives. Theoretically, the temporal information (Ca^2+^ oscillation frequency) can be converted into chemical information, such as protein phosphorylation cycles through Ca^2+^‐activated kinases and phosphatases (Salazar *et al*. 2008). Neural stem cells can decode such information to elicit cellular activities, including proliferation, transcriptional regulation, and synaptic transmission (Fig. 5E).

In the cerebral cortex, neural stem cells generate spontaneous calcium waves in a stage‐dependent manner, where late embryonic radial glia cells (E16–17 rat embryos) generate waves of a higher frequency and that travel over a longer distance than those generated by early radial glia cells (E12–13 embryos) (Owens & Kriegstein 1998; Weissman *et al*. 2004). These synchronous Ca^2+^ waves are propagated along adjacent neural stem cells through gap junctions and activate voltage‐gated Ca^2+^ channels in cell membranes. Disruption of Ca^2+^ oscillations using ATP receptor antagonists (Weissman *et al*. 2004) and gap junction inhibitors (Malmersjö *et al*. 2013) results in decreased BrdU labeling in neural stem cells. Notably, in the cerebral cortex, these gap junctions are also established between the daughter cells that arise from a common neural stem cell origin; this process generates ontogenic columns across the radial dimension that exhibit preferential electrical coupling (Yu *et al*. 2012). These results suggest that Ca^2+^ wave propagation and temporal patterns can act as a pacemaker of neural stem cell proliferation and connectivity.

Correlated Ca^2+^ activity also plays roles in establishing the output of neuronal subtypes during embryogenesis. In *Xenopus laevis*, Ca^2+^ spikes exhibit neuron‐specific patterns in the embryonic spinal cord, in which Rohon‐Beard neurons, dorsolateral interneurons and ventral motoneurons exhibit increased Ca^2+^ activity. These neurons also exhibit distinct frequency patterns throughout the developmental time course, in which the activity of Rohon‐Beard cells is constantly low, while dorsolateral interneurons exhibit monotonic increments and motoneurons show a low‐to‐high stepwise increase (Borodinsky *et al*. 2004). Suppressing Ca^2+^ spikes in these neurons throughout the overexpression of an inwardly rectifying potassium channel (Kir2.1) results in an increase in glutamatergic and cholinergic transmitter subtypes. Conversely, increasing Ca^2+^ spike frequency through the overexpression of voltage‐gated sodium channels (Na_v_2a) decreases excitatory neurotransmitter and increases inhibitory transmitter subtypes. In dissociated cells cultured at low cell densities, this neurotransmitter switching occurs as a function of frequency, indicating that Ca^2+^ oscillation frequency can act as a cell‐autonomous temporal code to drive the expression of neurotransmitters in specific cell types (Borodinsky *et al*. 2004). It remains to be explored whether neural specification in mammalian neural stem cells also involves temporal codes mediated by spontaneous Ca^2+^ oscillations.

## Linear temporal code‐driven sequence determination

Periodic shifts in molecular and cellular states play key roles in the fate decisions of neural stem cells. However, increasing evidence suggests that unidirectional changes in neuronal states over the developmental time course utilize linear temporal codes that switch cell identities through a ratchet‐like mechanism. Below, we discuss the molecular mechanisms of non‐periodic coding of developmental timers, which transmit time information in neural stem cells.

### Transcriptional network‐ and feedback‐driven temporal codes

Hierarchical cascades of gene networks triggered through interactions between transcription factors play fundamental roles in developmental sequence decisions (Fig. 5D, ‘*hierarchical cascade*’). Greater understanding of the gene cascades encoded in neural stem cells has been achieved for invertebrate systems. In *Drosophila*, the temporal progression of U1–U5 Even‐skipped (Eve)‐positive neurons, which sequentially express Hunchback (Hb) ‐> Kruppel (Kr) ‐> Pdm1 (Pdm) ‐> Castor (Cas) ‐> Grainyhead (Grh) transcription factors in both neuroblasts and their progeny, has been well characterized (Isshiki *et al*. 2001; Pearson & Doe 2003) (Fig. 2A). Manipulation of each of these transcription factors through extensive loss‐ and gain‐of‐function experiments has revealed the hierarchical cascade that occurs between these transcription factors: after the onset of *Hb* expression in neuroblasts, *Hb* is suppressed through cytokinesis (Grosskortenhaus *et al*. 2005), which triggers a sequential Kr‐> Pdm‐> Cas transcriptional cascade in a cell cycle‐ and cytokinesis‐independent manner. Therefore, downregulation of Hb may act as a timer to trigger Kr and downstream cascades to induce subsequent neuroblast production. When Hb is overexpressed in neuroblasts, the production of early‐born Hb^+^ neurons is prolonged at the expense of later‐born (Kr^+^, Pdm^+^, or Cas^+^) neurons. In this case, the ‘timer’ is what triggers Hb downregulation in the neuroblasts. For this, nuclear receptor protein seven‐up (SVP) was identified to transcriptionally repress Hb to cause a switch from Hb to Kr expression in neuroblasts (Kanai *et al*. 2005). SVP is transiently expressed in early neuroblasts, and in SVP misexpression mutants, Hb^+^ neuroblasts and their progeny are lost and are replaced by Kr^+^ neurons. What triggers SVP is unknown, although it does not require Hb expression (Kanai *et al*. 2005). Because Hb downregulation itself requires cytokinesis, the dilution of Hb over multiple rounds of cell divisions or feedback signals from newly generated neurons may contribute to this process (Kanai *et al*. 2005).

Transcriptional cascades also play a central role in neuronal production in the vertebrate CNS. In the cerebral cortex, neural progenitor cells generate CR cells, DL neurons, UL neurons, and glial cells in this temporal order (Frantz & McConnell 1996; Desai & McConnell 2000; Yu *et al*. 2012) (Fig. 2C). In this system, the forkhead transcription factor Foxg1 acts as a timer to evoke sequential DL and UL neurogenesis in neural progenitor cells, even when Foxg1 is induced during a later time period of cortical development (Kumamoto *et al*. 2013; Toma *et al*. 2014). Conversely, the loss of Foxg1 expression causes neural progenitor cells to acquire an early CR cell fate (Hanashima *et al*. 2004). Therefore, the expression of Foxg1 acts as a timer to trigger sequential temporal fate output in the cerebral cortex. In this case, Foxg1 expression itself is triggered by Fgf8 in the anterior neural ridge (ANR) (Suda *et al*. 1997; Kobayashi *et al*. 2002; Lagutin *et al*. 2003), which can pace the expansion of Foxg1 expression at a progressively more caudal level in the developing neural tube (Shimamura & Rubenstein 1997). The sequential temporal switch from DL to UL neurogenesis involves non‐cell‐autonomous feedback mechanisms; genetically ablating DL neurons during embryonic development results in an extended period of the generation of DL neurons by neural progenitor cells at the expense of UL neurogenesis. These results indicate that the sequential timing needed to terminate DL neurogenesis requires temporal cues that are produced by the DL neurons themselves (Toma *et al*. 2014) (Fig. 5B, bottom right). Indeed, this view is consistent with observations that neural stem cells induced from human or mouse ES/iPS cells are particularly sensitive to culture density; in particular, UL neurons can be induced at a higher probability in aggregated culture conditions (Eiraku *et al*. 2008; Gaspard *et al*. 2008; Kadoshima *et al*. 2013). Thus, post‐mitotic neurons act as a critical timing component in regulating neural stem cell fate decisions (Fig. 5B, bottom right).

Feedback‐dependent mechanisms are also observed in other neural systems. Taghert and colleagues were the first to perform laser ablation experiments in grasshopper embryos; these experiments demonstrated that the surrounding cells affect the cell fate of neuroblasts (Taghert *et al*. 1984). Similarly, in the retina, when early neural stem cells are co‐cultured with differentiated retinal cells of late‐stage origin, the capacity to differentiate into early‐generated amacrine cells was lost (Waid & McLoon 1998; Belliveau & Cepko 1999). Collectively, these results provide an emerging view of the linearly progressing timer, which is composed of a transcriptional regulatory network that segregates gene expression identities between temporally discrete subtypes, and neuron‐to‐stem cell feedback signaling, which breaks the network equilibrium to shift stem cells to the next temporally stable cell state.

### Threshold‐driven cumulative temporal codes (clepsydra, shishi‐odoshi)

Temporal regulation of cell identity through transcriptional mechanisms relies on cross‐repressive interactions and feedback networks, which establish gene expression states and trigger step‐wise changes in cell fate. In addition to these genetic networks, it has been demonstrated that epigenetic mechanisms also act as components of neural stem cell timers. In the *Drosophila* neuroblast NB7‐1 lineage, U1‐U5 Eve^+^ motor neurons are generated within the first five cell divisions. This is followed by the generation of 20 Eve^‐^ interneurons. Hb is expressed in neuroblasts and U1–U2 neurons; in Hb mutants, U1–U2 neurons are lost, indicating that Hb is a determinant of U1–U2 identity. Consistent with this, forced expression of Hb is sufficient to induce both U1 and U2 neurons. However, after the first five cell divisions, stem cells lose their ability to respond to Hb expression and acquire a motor neuron fate (Pearson & Doe 2003; Cleary & Doe 2006). These results indicate that the timer to switch from motor neuron to interneuron is set at five cell divisions. Using HA‐tagged Hb, Kohwi and colleagues demonstrated that the nuclear localization of the *Hb* gene locus changes from the nucleus center to the lamina by embryonic stage 12, which is the termination point of U5 neuron differentiation in NB7‐1 lineage. Forced ectopic localization of *Hb* gene loci to the nucleus center using *lamin* mutants (lamin^sz18^) is sufficient to induce motor neurons beyond U5 neuron production; therefore, epigenetic changes to gene loci over time appear to play a critical role in the temporal progression of neural stem cell output (Kohwi *et al*. 2013).

Epigenetic control of gene expression also plays prominent roles in establishing temporal cell fate in the cerebral cortex. In knockout mice that lack Polycomb group (PcG) expression, epigenetic control factors result in a delay in the switch from neurogenesis to gliogenic phase. Conversely, knockdown of high mobility group A (HMGA), a chromatin state modifier, results in a precocious switch from the neurogenic to the gliogenic phase (Hirabayashi *et al*. 2009; Kishi *et al*. 2012). PcG proteins bind to histone H3K27me3 marks and epigenetically represses gene expression. The accumulation of H3K27me3 histone marks is observed in the bHLH gene *Neurog1* as well as in the *HMGA2* locus. Therefore, cumulative binding of PcG proteins can act as a *clepsydra (shishi‐odoshi)*‐like timer; upon reaching a threshold, it results in constitutive and irreversible repression of neurogenic genes and triggers gliogenesis (Hirabayashi *et al*. 2009; Kishi *et al*. 2012) (Fig. 5D, ‘*cumulative signal*’). Together, histone modification‐mediated epigenetic mechanisms serve as a cell internal clock to count time in neural stem cells. Together with transcriptional cascades and feedback‐driven codes, neural stem cells decode the external and internal timers to produce neuronal output with the correct timing through the ratcheting of cell fates. Both quantitatively and qualitatively, questions remain concerning the nature of such an intrinsic timer, including how the speed of the histone binding events is physically controlled and how PcG proteins recognize specific loci within the genome. Addressing these questions will also explain whether the physical binding events vary from species to species, which would thereby contribute to a species‐specific *‘intrinsic timer’* of neurogenesis; this idea merits further investigation.

## Temporal scaling of neurogenesis in the CNS

The studies discussed above reveal the fundamental role of temporal cues in determining stem cell output within each neural development system. However, within an organism, different CNS regions exhibit variable timing with respect to the entry and exit of the neurogenesis phase during development. Here, we highlight features of the temporal scaling of neurogenesis in the nervous system; such features are critical parameters in determining tissue‐specific neuronal output.

In the mouse CNS, the first neural stem cells are identified as Sox1‐positive primitive neuroepithelial cells at E7.5, prior to neural tube formation. At E8.25, neural stem cells start to express regional identity markers, including Foxg1, Otx2, and Emx2. Neural tube formation commences at approximately E8.5 and is paralleled by regionalization across the rostrocaudal axis through multiple morphogens: Fgf8, Wnt1, and Wnt3a in the hindbrain; and Fgf8, BMP2/4, and Shh in the telencephalon and diencephalon. These morphogens trigger neurogenesis in a caudal‐to‐rostral temporal order. In the spinal cord, neurogenesis starts as early as E9.5; these are the first neurons of CNS development. In turn, forebrain neural stem cells called radial glia cells are identified at E10.5, and neurogenesis in the retina is initiated after optic cup formation at approximately E11.5. The anterior neural tube (the telencephalon and diencephalon) shows delayed neurogenesis onset due to the response to BMPs, Wnts and retinoic acid signaling. These temporal shifts in neurogenesis also occur along the dorsoventral axis; the trend is toward earlier differentiation in the ventral neural tube and later differentiation in the dorsal neural compartments. In this case, the higher Wnt concentration and signaling in the dorsal neural tube augments the stem cell properties of the neural progenitors, resulting in earlier differentiation of the ventral progenitors. In the telencephalon, BLBP‐expressing radial glia cells are present throughout the telencephalon; however, dorsal and ventral telencephalic stem cells also exhibit different neurogenesis windows. Isolated cortical and ganglionic eminence radial glia show different neurogenic abilities, and cortical RGCs initiate and terminate neurogenesis later than ganglionic eminence radial glia (Anthony *et al*. 2004).

The termination of neurogenesis is defined by the loss of the potential for neuron generation in stem cells, which is accompanied by the onset of gliogenesis. The transition from neurogenesis to gliogenesis also shows temporal shifts along the rostrocaudal axis; the spinal cord shows the earliest gliogenesis transition, at E12.5 in the mouse, whereas in the cerebral cortex, gliogenesis only occurs after E16.5. In the retina, Muller glia cells, which are trophic supporting cells, also appear the latest—at approximately P0. In these regions, the onset of gliogenesis is tightly coupled with neurogenesis; therefore, manipulation of the neurogenesis period is often accompanied by a shift in the timing of gliogenesis (Hirabayashi *et al*. 2009; Seuntjens *et al*. 2009). However, there is a minor population of neural stem cells that maintains neurogenic potential until the postnatal stage, during which they contribute to postnatal neurogenesis (Fuentealba *et al*. 2015; Furutachi *et al*. 2015). These observations suggest that while the neurogenic timer is controlled in a tissue‐ and lineage‐specific manner, there are minor populations of neural stem cells that are refractory to temporal cues and pace their neurogenic timing. How these neural stem cells of embryonic origin control their ‘lineage‐specific clock’ and how they are activated at postnatal stages is still unknown.

The timer that sets the start and end of the neurogenic period are critical determinants of neuronal number and ultimate brain size; therefore, how such temporal scaling is controlled among different species is an intriguing question. Is the neurogenic period scaled proportionally to the developmental time scale? Among avian species, chickens and ducks have identical neurogenic periods during development and exhibit similar growth curves, but the telencephalon volume of duck embryos is greater than that of chick embryos at early developmental stages. This is due to the accelerated expansion of neural stem cell population during early duck embryogenesis (Charvet & Striedter 2009). Similar differences have been reported between chickens and quails, which indicates that neurogenesis is triggered according to a species‐specific timer that is set at a relatively early stage of development (Charvet & Striedter 2010). Heterotopic transplantation of zebra finch stem cells into early‐stage quail embryos showed that the transplanted cells contributed a bigger proportion of neurons in quail than in zebra finch (Chen *et al*. 2012), suggesting that the quail brain niche affected the neurogenesis of the zebra finch transplants via an extrinsic mechanism.

Within a broader amniotes clade, the temporal scaling of neurogenesis also exhibits a species‐specific time scale (Nomura *et al*. 2013). Analysis of neurogenesis and cell cycle speed in mouse, chick, turtle and gecko revealed that neurogenesis in the brain occupies approximately one‐third of the entire embryonic period in amniotes. With this fixed time window, cell cycle frequency and the timing of switch from self‐renewal to neurogenesis become critical parameters for determining the final neuronal number. Among these species, the gecko exhibits an extremely slow cell cycle (i.e., low frequency), resulting in a smaller cell population and brain size. The onset of neurogenesis appears later in mouse than in chicken and gecko, indicating that mammals undergo specific symmetric expansion of neural stem cells prior to the onset of neurogenesis. Indeed, once neural stem cells enter the asymmetric division phase, there is a point of no return, in which the output of neurons from a single neural stem cell is predictable (Gao *et al*. 2014).

## Perspectives

Neuroanatomical, biochemical and physiological experiments have indicated that temporal codes play fundamental roles in regulating coordinated neural stem cell behavior during developmental events. However, these findings have not answered questions concerning temporal coding and neural stem cells. First, compared to other cell types, are neural stem cells particularly prone to temporal codes generated intrinsically and extrinsically to the system? It is possible that the highly correlated cell type‐specific output linked to ‘time’ was easily visible by early neuroanatomists and that recently discovered oscillations in gene expression and spontaneous activity biased the identification of temporal pattern‐dependent regulation of developmental events specifically in the nervous system. However, it is also plausible that the need to sense and respond to external physical cues at multimodal auditory, visual, and motor levels with high temporal precision, may have adapted stem cells of these origins to respond to molecular and cellular cues early in their developmental and evolutionary ontogeny. Second, does an ‘absolute clock’ really exist in development? Could we manipulate multiple parameters that change the timing of distinct events without affecting the ostensibly intrinsic timer? Indeed, it was Einstein who proposed that even in the physical world, not just motion but also time and space are relative, and who challenged the Newtonian view of the presence of ‘absolute time,’ which in theory should flow equally without reference to anything external.
